# Epidemiology and Clinical Profiles of *Bacteroides fragilis* Bacteremia in a Tertiary Care Setting: A 5‐Year Review

**DOI:** 10.1155/cjid/5844253

**Published:** 2026-06-21

**Authors:** Meliha Çağla Sönmezer, Enes Erul, Nagihan Aka Türkmen, Taha Koray Sahin, Mervenur Demir Cuha, Gülşen Hazırolan, Murat Akova

**Affiliations:** ^1^ Infectious Diseases and Clinical Microbiology Department, Hacettepe University Faculty of Medicine, Ankara, 06100, Turkey, hacettepe.edu.tr; ^2^ Internal Medicine Department, Hacettepe University Faculty of Medicine, Ankara, 06100, Turkey, hacettepe.edu.tr; ^3^ Microbiology and Clinical Microbiology Department, Hacettepe University Faculty of Medicine, Ankara, 06100, Turkey, hacettepe.edu.tr

**Keywords:** anaerobic infections, antibiotic resistance, *Bacteroides fragilis*, mortality risk factors, nosocomial infections

## Abstract

**Background:**

Anaerobic infections remain clinically significant yet underrecognized due to diagnostic challenges and limited routine susceptibility testing in clinical laboratories. Among these pathogens, *Bacteroides fragilis* is a clinically important anaerobe associated with bloodstream and deep‐seated infections. This study aimed to evaluate the epidemiology, clinical characteristics, and antimicrobial resistance patterns of *Bacteroides fragilis* bacteremia in a tertiary care hospital.

**Materials and Methods:**

This retrospective study included patients with blood cultures positive for *Bacteroides fragilis* between July 2018 and January 2023. Demographic characteristics, clinical features, infection sources, treatment regimens, and antimicrobial resistance patterns were analyzed.

**Results:**

Our analysis found a higher rate of *Bacteroides fragilis* bacteremia among cancer patients, likely linked to cancer treatments that compromise gastrointestinal defenses. Notably, both hospital‐acquired infection (OR: 4.99, 95% CI: 1.3–19.07, *p* = 0.019) and respiratory tract infection (OR: 8.19, 95% CI: 1.21–55.35, *p* = 0.031) were associated with an increased risk of death within 30 days, highlighting the importance of vigilance in healthcare environments. Resistance to clindamycin was substantial, while ampicillin resistance was expected because of the intrinsic resistance profile of Bacteroides species. While carbapenem resistance was uncommon, its presence signals a shifting resistance pattern. Metronidazole remained effective, supporting its continued use.

**Conclusion:**

*Bacteroides fragilis* bacteremia is associated with significant mortality, particularly in nosocomial settings. Continuous surveillance and appropriate antimicrobial stewardship are essential for effective management.

## 1. Background

Anaerobic bacteria constitute a major component of the normal human microbiota, particularly colonizing the gastrointestinal tract, oral cavity, female genital tract, and skin. Under physiological conditions, these microorganisms exist in a commensal relationship with the host and contribute to microbial homeostasis. However, disruption of mucosal barriers due to surgery, trauma, malignancy, or immunosuppression may facilitate translocation of anaerobic bacteria into normally sterile sites, leading to severe and potentially life‐threatening infections. Despite their clinical importance, anaerobic infections are often underdiagnosed due to challenges in specimen collection, delayed growth in culture, and the limited availability of routine susceptibility testing in many clinical laboratories [1, 2].

*Bacteroides fragilis* (*B. fragilis*) is considered one of the most clinically significant anaerobic pathogens and is frequently implicated in bloodstream infections, intra‐abdominal infections, and abscess formation. Its pathogenicity is largely attributed to several virulence factors, including capsular polysaccharides that facilitate immune evasion and promote abscess formation. In addition to its role as an opportunistic pathogen, *B. fragilis* exhibits considerable biological and pathogenic diversity [3, 4].

Certain strains of *B. fragilis*, including enterotoxigenic (ETBF) and nontoxigenic (NTBF) variants, differ in their pathogenic potential. ETBF strains produce *B. fragilis* toxin, a metalloprotease associated with inflammatory diarrheal disease and colorectal carcinogenesis, whereas NTBF strains are more commonly involved in invasive opportunistic infections [5]. These differences highlight the dual role of *B. fragilis* as both a commensal organism and a clinically important pathogen [3–5].

In recent years, increasing antimicrobial resistance among *B. fragilis* isolates has emerged as a significant concern, complicating empirical treatment strategies and clinical management. Resistance to commonly used agents such as clindamycin and beta‐lactam antibiotics has been widely reported, while carbapenem resistance, although less frequent, is of clinical importance due to its association with the cfiA gene and potential for rapid dissemination [2, 6, 7].

While awaiting susceptibility testing results, empirical therapy plays a pivotal role in shaping clinical and microbiological outcomes, although the literature is conflicting [8, 9]. Unfortunately, there are no universally accepted guidelines governing the treatment of *B. fragilis* infections, and only a limited number of recent studies have addressed this issue [6, 10, 11]. Understanding the nuances of antibiotic susceptibilities is crucial, as *B. fragilis is* increasingly demonstrating antibiotic resistance, necessitating strict antibiotic stewardship practices [9, 12–14].

Although several studies have evaluated anaerobic bacteremia and Bacteroides species, data focusing specifically on *B. fragilis* bacteremia and its clinical characteristics remain limited, particularly at the single‐center level. Therefore, this study aims to evaluate the epidemiological characteristics, clinical features, and antimicrobial resistance patterns of *B. fragilis* bacteremia over 5 years in a tertiary care center [1, 6, 7].

## 2. Methods

### 2.1. Patients

This retrospective study included patients with blood cultures positive for *Bacteroides fragilis* treated at Hacettepe University Hospital between July 2018 and January 2023. This medical facility is a tertiary care institution with more than 950 beds, encompassing all medical specialties, an emergency department, and an intensive care unit (ICU). Requests for cultures were initiated by attending physicians who determined the patient’s diagnosis and subsequent treatment plan.

The gathered data included demographic information such as age and gender, confirmation of *B. fragilis* as the causative organism based on MALDI‐TOF‐MS identification, quick sequential organ failure assessment (qSOFA) scores, presence of fever, categorization of infection as either nosocomial or community‐acquired, identification of relevant risk factors or underlying medical conditions, pertinent laboratory findings, history of prior surgeries, presence of any form of cancer and/or receipt of cytotoxic treatments, and previous administration of antimicrobial agents. Additionally, comprehensive information regarding clinical manifestations, the clinical setting of pathogen isolation (including medical or surgical wards, ICUs, oncology or hematology wards, and outpatient settings), prescribed treatment regimens, and ultimate patient outcomes was meticulously documented.

### 2.2. Microbiology: Isolation, Identification, and Antibiotic Susceptibility Testing of Strains

A total of 76 nonduplicate *Bacteroides fragilis* blood culture isolates were included. In our hospital, at least two blood culture sets are obtained from adult patients to evaluate bloodstream infections. These sets are drawn from different peripheral sites, allowing discrimination between true bacteremia and contamination. Microbiological findings were interpreted together with the clinical presentation to distinguish true bacteremia from contamination.

All isolates were identified using MALDI‐TOF MS (Microflex, Bruker Daltonics, Bremen, Germany) in linear positive mode. A match score of ≥ 2.0 was considered reliable for species‐level identification. The *B. fragilis* ATCC 25285 strain was used as a reference strain [15].

Antimicrobial susceptibility testing was performed using the reference agar dilution method (bioMérieux, France) on Brucella agar plates supplemented with 5% defibrinated sheep blood, hemin (5 µg/mL), and vitamin K (1 µg/mL), in accordance with the Clinical and Laboratory Standards Institute (CLSI) guidelines [16]. Plates were incubated in anaerobic jars (Oxoid AnaeroJar, Thermo Fisher Scientific, USA) with BD GasPak EZ anaerobe gas‐generating sachets at 37°C for 48 h. Minimum inhibitory concentrations (MICs) were determined and interpreted according to the CLSI M100‐Ed33 breakpoints [17].

### 2.3. Clinical Definitions

*Bacteroides fragilis* bacteremia was defined as the isolation of *B. fragilis* from at least one blood culture in a clinically compatible setting. Infections were classified as nosocomial or community‐acquired based on the timing of culture positivity.

Episodes were classified as monomicrobial when only *B. fragilis* was isolated and as polymicrobial when two or more distinct noncontaminating microorganisms were isolated within 7 days of the first positive culture. Infection was categorized as nosocomial if positive cultures were acquired 48 h or more after the patient’s admission to the hospital. Conversely, it was considered community‐acquired if *B. fragilis* was isolated within 48 h of admission, and the patient had not been hospitalized in the preceding 2 weeks. A focus infection was established when at least two of the following criteria were met: isolation of bacteria from a focal culture, presence of radiological or clinical signs indicative of a focal infection, and the manifestation of symptoms consistent with a focal infection. A primary infection was identified in cases where no specific infectious site could be determined, but the blood cultures tested positive for *B*. *fragilis*, and the patient exhibited clinical signs of infection.

Several predisposing factors for infection were considered, including recent surgical procedures requiring general anesthesia or cytotoxic agents within the past month, preceding the onset of infection, and the presence of cancer, diabetes mellitus, and chronic illnesses. The patient’s condition’s severity was assessed using the qSOFA, which involves alteration of the consciousness level (Glasgow score < 13) (1 point), systolic blood pressure ≤ 100 mmHg (1 point), and respiratory rate ≥ 22 bpm (1 point) [18]. Septic shock was defined by the presence of severe sepsis accompanied by persistent hypotension necessitating vasopressors to maintain a mean arterial pressure of 65 mmHg and having a serum lactate level exceeding 2 mmol/L (18 mg/dL) despite sufficient volume resuscitation [18]. All these clinical parameters were recorded on the day when the initial positive culture was obtained. Time‐to‐positivity (TTP), a proxy for bacterial load and microbial growth rate, was defined as the duration between the commencement of incubation and the detection of bacterial growth by an automated system (first positive culture vial). Mortality information was tracked up to the 30th day after the commencement of the episode.

### 2.4. Statistical Analysis

Continuous variables were summarized using either mean ± standard deviation (SD) or median with interquartile range (percentiles 25 and 75), depending on the distribution’s normality, assessed via Shapiro–Wilk’s tests. Discrete variables were presented as absolute counts and percentages. Comparisons between two independent groups were conducted using Student′s *t*‐tests (or Welch’s test in cases of unequal variances) or Mann–Whitney *U* tests as appropriate. Proportional comparisons were evaluated using chi‐squared tests or Fisher’s exact tests, depending on the situation. To ascertain potential predictors of mortality, a multivariable logistic regression model was executed. Subsequently, a backward stepwise selection method was employed. At each step, the likelihood ratio test was used to evaluate the contribution of each variable to the model in a statistically significant manner. Survival curves stratified by infectious site and acquisition of infection were generated utilizing the Kaplan–Meier method and compared using the log‐rank test. In all analyses, a *p*n value of ≤ 0.05 was considered statistically significant. Data analysis was conducted using SPSS software version 18. This study was performed in compliance with the principles outlined in the Declaration of Helsinki. This study was approved by the Hacettepe University Health Sciences Research Ethics Committee (Approval No: SBA 23/410; Decision No: 2023/08‐28).

## 3. Results

### 3.1. Demographic Characteristics of the Patients With *Bacteroides fragilis* Bacteremia

The mean age of patients with *B*. *fragilis* bacteremia was 57.8 ± 21.4 years, with 42 (55.3%) of them being females. Among these cases, 28 (36.8%) occurred in surgical wards, 16 (21.1%) in ICUs, 14 (18.4%) in hemato‐oncology facilities, 9 (11.8%) in emergency units, and 9 (11.8%) in medical wards. Out of the total patients, 33 (43.4%) were classified as community‐acquired, while the remaining 43 (56.6%) were designated as hospital‐acquired.

Many patients presented with significant underlying illnesses or conditions during the onset of infection, particularly with solid cancer (*n* = 34, 44.7%), recent surgical procedures (*n* = 24, 31.6%), and the use of antimicrobials within 30 days before infection (*n* = 38, 50%). The predominant comorbidities observed were diabetes (*n* = 17, 22.4%), chronic obstructive pulmonary disease (COPD) (*n* = 10, 13.2%), transient ischemic attack (TIA) or cerebrovascular accident (CVA) (*n* = 8, 10.5%), congestive heart failure (*n* = 5, 6.6%), and hemodialysis (*n* = 6, 7.9%). The median Charlson comorbidity index (CCI) of all patients was 4.5 [2–7], and there was no significant difference between the community‐acquired and nosocomial‐acquired groups (*p* = 0.097). Table 1 summarizes the clinical characteristics of the patients in detail.

**TABLE 1:** Clinical characteristics of patients with *Bacteroides fragilis* bacteremia (nosocomial vs. community‐acquired).

	**Total (*n* = 76)**	**Nosocomial (*n* = 43)**	**Community (*n* = 33)**	**p** **value for univariate comparison**

Male, *n* (%)	34 (44.7%)	20 (46.5%)	14 (42.4%)	0.72
Age, mean (SD) (years)	57.8 (21.4)	59.6 (21.4)	55.5 (21.5)	0.41
Clinical findings				
Fever	37 (48.7%)	19 (44.2%)	18 (54.5%)	0.588
Digestive symptoms	30 (39.5%)	19 (44.2%)	11 (33.3%)	
Respiratory symptoms	4 (5.3%)	3 (7%)	1 (3%)	
Neurological symptoms	5 (6.6%)	2 (4.7%)	3 (9.1%)	
Laboratory results				
CRP level (mg/dL), median (IQR)	16.1 (7.8–26.6)	15.4 (6.52–22.6)	20.2 (10.4–28.3)	0.125
Pct level (μg/L), median (IQR)	1.17 (0.36–4.46)	1.29 (0.3–4.43)	0.96 (0.36–4.66)	0.413
WBC level/microliters, median (IQR)	11.600 (8200–14.400)	10.500 (7100–14.100)	13.350 (9975–17.975)	0.053
Days between TTP and death, median (IQR)	16.5 (4.7–52)	7.5 (3.7–38)	45.5 (13.2–60.7)	**0.055**
Length of stay, median (IQR), days	25.5 (12–51.75)	38 (19–69)	14 (4.5–32.5)	**0.001**
Charlson score, median (IQR)	4.5 (2–7)	6 (3–7)	3 (1–6.5)	0.097
Hemodialysis, *n* (%)	6 (7.9%)	4 (9.3%)	2 (6.1%)	0.692
Diabetes, *n* (%)	17 (22.4%)	10 (23.3%)	7 (21.2%)	0.833
Congestive heart failure, *n* (%)	5 (6.6%)	3 (7%)	2 (6.1%)	0.874
Surgery within 30 days before hospitalization, *n* (%)	24 (31.6%)	18 (41.9%)	6 (18.2%)	**0.028**
Solid neoplasia, *n* (%)	34 (44.7%)	24 (55.8%)	10 (30.3%)	**0.028**
Hematological neoplasia, *n* (%)	1 (1.3%)	1 (2.3%)	0 (0%)	0.381
CRP, median (IQR) (mg/L)	161 (78.1–265.5)	154 (65.2–226)	202.5 (104–283.2)	0.125
qSOFA, median (IQR)	0 (0–1)	0 (0–1)	0 (0–1)	0.120
Septic shock, *n* (%)	30 (39.5%)	21 (48.8%)	9 (27.3%)	0.057
ICU, *n* (%)	27 (35.5%)	20 (46.5%)	7 (21.2%)	**0.022**
Mortality at 30 days, *n* (%)	16 (21.3%)	13 (31%)	3 (9.1%)	**0.044**
Polymicrobial infection, *n* (%)	35 (46.1%)	19 (44.2%)	16 (48.5%)	0.709
Duration of antibiotic therapy, median (IQR) (days)	14 (7.2–17.7)	11 (7–17)	15 (10–18)	**0.001**
Adequate empirical antibiotic therapy, *n* (%)	64 (84.2%)	41 (95.3%)	23 (69.6%)	**0.002**
Origin of infection				
Abdominal, *n* (%)	27 (35.5%)	17 (39.5%)	10 (30.3%)	0.186
Surgical wounds and osteoarticular, *n* (%)	12 (15.8%)	3 (7%)	9 (27.3%)	
Primary, *n* (%)	24 (31.6%)	14 (32.6%)	10 (30.3)	
Pulmonary, *n* (%)	4 (5.3%)	3 (7%)	1 (3%)	
Urinary and genital, *n* (%)	8 (10.5%)	6 (14%)	2 (6.1%)	
Ear–nose–throat, *n* (%)	1 (1.3%)	0 (0%)	1 (3%)	

*Note:* Bold values indicate statistical significance (*p* < 0.05).

### 3.2. Clinical Outcomes of the Patients With *Bacteroides fragilis* Bacteremia

Septic shock was present in 30 patients (39.5%), with 27 patients (35.5%) admitted to the ICU. ICU admission was more prevalent in the nosocomial group (46.5% vs. 21.2%, *p* = 0.022). Of the entire cohort, 53 patients (69.7%) demonstrated positive clinical outcomes and were subsequently discharged from the healthcare facility, whereas 23 (30.3%) patients died. Among the deceased patients, most deaths were infection‐related; however, given the high burden of comorbidities in this population, the exact attribution of mortality remains uncertain.

**FIGURE 1:**
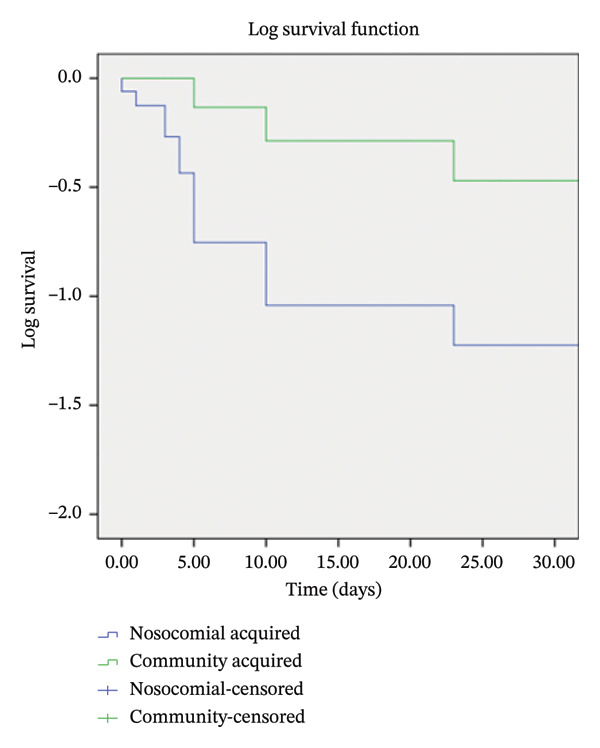
Kaplan–Meier curves for the 30‐day survival according to the acquisition (nosocomial vs. community‐acquired).

The nosocomial group exhibited a longer median hospital stay compared to the community group (38 (19–69) vs. 14 (4.5–32.5) days, *p* = 0.001). Additionally, the interval between time to positivity and death was notably shorter in the nosocomial group (7.5 (3.7–38) vs. 45.5 (13.2–60.7)) but did not reach statistical significance (*p* = 0.055). In the univariate analysis (Table 2), the nosocomial group had poorer outcomes for 30‐day mortality (OR: 3.21 (0.91–11.37), *p* = 0.044) (Figure 1). In the Cox proportional hazard model analysis (Table 2), the nosocomial group (OR: 4.99 (1.3–19.07), *p* = 0.019) remained an independent risk factor for 30‐day mortality.

**TABLE 2:** Mortality analysis in patients with *Bacteroides fragilis* bacteremia.

Covariate	Level of the covariate	Univariate analysis		Multivariable cox model	
		HR (95% CI)	*p* value	HR (95% CI)	*p* value
Gender	Male Female	1 0.72 (0.26–1.95)	0.502	—	—

Age	< 65 (Ref) ≥ 65	1 0.70 (0.25–1.93)	0.469	0.81 (0.19–3.47)	0.781

Charlson score (CCI)	CCI < 4 (Ref) CCI ≥ 4	1 0.58 (0.16–2.05)	0.399	0.57 (0.10–3.04)	0.517

Acquisition of infection	Community (Ref) Nosocomial	1 3.21 (0.91–11.37)	**0.044**	4.99 (1.3–19.07)	**0.019**

Surgery within past 30 days	No (Ref) Yes	1 0.65 (0.21–2.04)	0.466	—	—

Patients admitted in ICU	No (Ref) Yes	1 1.16 (0.37–3.62)	0.779	1.11 (0.29–4.80)	0.807

Septic shock	No (Ref) Yes	1 0.57 (0.13–2.53)	0.440	2.24 (0.43–11.66)	0.338

Origin of infection	Abdominal (Ref)	1	0.272		0.083
	Primary	2.1 (0.44–9.93)	0.347	2.35 (0.49–11.23)	0.281
	Pulmonary	5.6 (0.90–34.80)	**0.064**	8.19 (1.21–55.35)	**0.031**
	Urogenital	1.6 (0.27–9.74)	0.594	0.96 (0.15–5.88)	0.966

*Note:* Bold values indicate statistical significance (*p* < 0.05).

### 3.3. Presumed Primary Sources of *Bacteroides fragilis* Bacteremia

**FIGURE 2:**
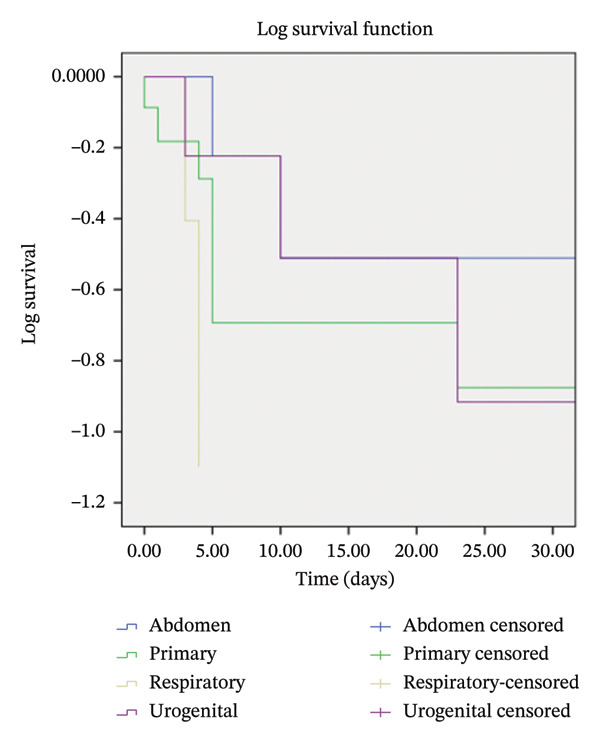
Kaplan–Meier curves for the 30‐day survival according to the primary source of infection.

The primary origins of *B*. *fragilis* bacteremia included abdominal sources (*n* = 27, 35.5%), primary sources (*n* = 24, 31.6%), surgical wounds and osteoarticular sources (*n* = 12, 15.8%), urinary and genital sources (*n* = 8, 10.5%), and respiratory sources (*n* = 4, 5.3%). The mortality rates for the 30‐day survival, categorized by the infectious site, were as follows: respiratory (*n* = 3, 75%), primary (*n* = 8, 33.3%), urogenital (*n* = 3, 37.5%), abdominal (*n* = 2, 7.4%), ear–nose–throat (*n* = 0, 0%), and osteoarticular and surgical wounds (*n* = 0, 0%). Patients with pulmonary infection had the highest mortality rate but did not reach statistical significance in univariate analysis (OR: 5.6 (0.9–34.80), *p* = 0.066) (Figure 2). In the Cox proportional hazard model analysis (Table 3), pulmonary infection (OR: 8.19 (1.21–55.35), *p* = 0.031) emerged as an independent risk factor for 30‐day mortality.

**TABLE 3:** Prevalence of resistance to different antimicrobial agents of *Bacteroides fragilis* isolates over 5 years.

Antimicrobial agent	2018	2019	2020	2021	2022	*p* value
Metronidazole	0/13 (0%)	0/15 (0%)	0/16 (0%)	0/20 (0%)	0/12 (0%)	0.390
Tigecycline	1/13 (7.7%)	2/15 (13.3%)	0/16 (0%)	1/20 (5%)	0/12 (0%)	< 0.001
Piperacillin–tazobactam	4/13 (30.8%)	1/15 (6.7%)	1/16 (6.3%)	2/20 (10%)	1/12 (8.3%)	0.049
Meropenem	3/13 (23.1%)	2/15 (13.3%)	3/16 (18.8%)	2/20 (10%)	0/12 (0%)	0.470
Clindamycin	7/13 (53.8%)	5/15 (33.3%)	4/16 (25%)	9/20 (45%)	1/12 (8.3%)	< 0.001
Ampicillin	13/13 (100%)	14/15 (93.3%)	16/16 (100%)	20/20 (100%)	11/12 (91.7%)	0.302

### 3.4. Antimicrobial Treatment and Resistance Profiles of *Bacteroides fragilis* Blood Culture Isolates

We observed that 64 cases (84.2%) received appropriate empirical treatments, involving a total of 45 patients (59.2%) with a single antibiotic, while 19 patients (25%) were administered two or more antibiotics. The primary antimicrobial of choice was piperacillin–tazobactam (*n* = 15, 19.7%), followed by meropenem (*n* = 13, 17.1%), ampicillin/sulbactam (*n* = 10, 13.2%), and ceftriaxone (*n* = 10, 13.2%) as per the physician’s discretion. In the community group, patients underwent a more extended course of antibiotic therapy compared to those in the nosocomial group (15 [10–15, 18–20] vs. 11 [7–15, 18, 19] days, *p* = 0.001).

Metronidazole showed the most favorable susceptibility profile, and no resistance was detected among the isolates. Tigecycline (5.3%), piperacillin–tazobactam (11.8%), and meropenem (13.2%) also demonstrated relatively low resistance rates. In contrast, clindamycin resistance was substantial (34.2%), while ampicillin resistance was the highest (93.4%). Trends in annual resistance rates are presented in Table 3.

### 3.5. Concomitantly Isolated Bacteria

Polymicrobial infections accounted for 46% of clinically significant cases. The prevalent species included *Escherichia coli*, *Enterococcus* spp., *Klebsiella* spp., and *Streptococcus anginosus*. The distribution of aerobic pathogens in polymicrobial infections is outlined in Table 4.

**TABLE 4:** Distribution of the concomitantly isolated aerobic bacteria.

Concomitantly isolated aerobes	*n* (%)
*E. coli*	12 (15.8%)
*Klebsiella pneumoniae*	5 (6.6%)
*Enterococcus faecium*	5 (6.6%)
*Enterococcus faecalis*	4 (5.3%)
*Streptococcus anginosus*	4 (5.3%)
*Staphylococcus aureus*	3 (3.9%)
*Staphylococcus epidermidis*	3 (3.9%)
*Pseudomonas aeruginosa*	2 (2.6%)
*Klebsiella oxytoca*	2 (2.6%)
Total	35 (46%)

## 4. Discussion

*B. fragilis* is often underdiagnosed due to the inherent challenges associated with anaerobic microbiology, including slow bacterial growth, oxygen sensitivity, and difficulties in specimen collection and transport. From a mechanistic perspective, antimicrobial resistance in *B. fragilis* is mediated by multiple pathways, including constitutive beta‐lactamase production, cfiA gene‐associated carbapenem resistance, and the presence of mobile genetic elements that facilitate horizontal gene transfer [5, 9]. Furthermore, gene expression may be regulated by insertion sequences, leading to variability between phenotypic and genotypic resistances. These complex microbiological and molecular characteristics contribute to both diagnostic uncertainty and therapeutic challenges in the management of *B. fragilis* infections [2, 4, 11]. Understanding these microbiological characteristics is essential for interpreting the clinical findings of our study.

Building on these microbiological insights, we conducted a comprehensive clinical assessment of patients with *B. fragilis* bacteremia and followed them until hospital discharge or death. Several clinical differences between nosocomial and community‐acquired infections observed in our study deserve further consideration. Patients with nosocomial infection had significantly longer hospital stays, higher rates of ICU admission, and a higher frequency of recent surgery and malignancy. These findings likely reflect increased exposure to invasive procedures, disruption of mucosal barriers, and a higher burden of immunosuppression in hospitalized patients, all of which are well‐recognized risk factors for bloodstream infections [16, 19, 20]. This is consistent with the higher rates of ICU admission and recent surgical interventions observed in the nosocomial group.

*B. fragilis* bacteremia showed an increase among patients with malignancy, with almost half of the patients identified as having malignant conditions. This escalation might be attributed to the heightened utilization of anticancer therapies impacting the gastrointestinal barrier and potentially serving as an entry point [17]. Polymicrobial infection accounted for 46% of cases in our study, with *E*. *coli* and *Klebsiella pneumoniae* being the pathogens most detected in cultures containing *B*. *fragilis*. This high rate of polymicrobial infection may complicate clinical interpretation and delay targeted therapy.

*B. fragilis* bacteremia has been associated with substantial mortality, exceeding 19% in several studies of anaerobic bacteremia (22, 23, 26). In our study, nosocomial acquisition and respiratory source of infection were independently associated with 30‐day mortality. Distinguishing whether mortality is directly attributable to *B. fragilis* bacteremia or to underlying comorbid conditions is inherently challenging. In our cohort, most patients who died had significant underlying diseases, suggesting that mortality likely reflects a combination of infection severity and host‐related factors rather than a single causative mechanism, which is consistent with previous studies on anaerobic bacteremia [19, 21]. The high mortality associated with respiratory tract infections may reflect aspiration events, delayed initiation of anaerobic coverage, and the presence of severe underlying diseases, particularly in critically ill patients. Additionally, patients with nosocomial infection experienced an extended duration of hospitalization. The 30‐day mortality rate observed in our cohort is consistent with previously reported rates ranging between 15% and 30% in anaerobic bacteremia. Similar studies have demonstrated that hospital‐acquired anaerobic infections are associated with higher mortality, often related to delayed appropriate therapy and increased comorbidity burden [1, 18, 19, 21–23].

The susceptibility of anaerobes to antibiotics has undergone substantial transformations, shifting from profiles characterized by complete susceptibility to the emergence of multidrug resistance. *B*. *fragilis* exhibits the highest rates of antibiotic resistance among anaerobes [7, 12, 13, 16, 23, 24]. Clindamycin showed elevated resistance rates in our study population. The high resistance rate to ampicillin observed in our study is expected, as Bacteroides species are known to be intrinsically resistant to many beta‐lactam antibiotics due to beta‐lactamase production [1, 2]. Our study did identify one multidrug‐resistant (MDR) isolate, characterized by acquired nonsusceptibility to clindamycin, meropenem, and tigecycline. Although carbapenem resistance remained relatively uncommon in our cohort, the detection of meropenem‐resistant isolates is clinically relevant. Carbapenem resistance in *B. fragilis* is most mediated by the cfiA gene, which encodes a metallo‐beta‐lactamase and may be activated by insertion sequences, leading to variable expression levels. In the present study, molecular characterization of resistance mechanisms, including detection of the cfiA gene, was not performed, which represents an important limitation. Therefore, we cannot determine whether the observed reduced susceptibility to meropenem was due to cfiA‐mediated resistance or to alternative mechanisms. Previous studies have demonstrated that cfiA‐positive isolates may remain phenotypically susceptible until gene expression is induced, highlighting the complexity of resistance detection and the potential for underestimation in routine clinical practice. These findings underscore the importance of incorporating molecular diagnostics into surveillance studies of *B*. *fragilis,* particularly in the context of emerging carbapenem resistance [9, 14, 24].

Metronidazole remains a viable choice, with a resistance rate of 0%, a positive aspect for antimicrobial susceptibility among the observed *B*. *fragilis* strains. When compared with previously published studies focusing on bloodstream isolates, the resistance rates observed in our cohort are largely consistent with global trends, particularly regarding high resistance to clindamycin and preserved susceptibility to metronidazole. However, variations in carbapenem resistance rates highlight the importance of local surveillance data [8, 13, 16, 19, 25]. When resistance patterns were evaluated over the 5‐year study period, no consistent increasing trend was observed for most antimicrobial agents. Metronidazole resistance remained absent throughout the study, supporting its continued role as a reliable treatment option. In contrast, clindamycin resistance showed significant variability over time, with fluctuating rates across years (*p* < 0.001), suggesting instability in resistance patterns rather than a steady increase. Similarly, piperacillin‐tazobactam resistance demonstrated a decreasing trend over time (*p* = 0.049), while meropenem resistance showed variability without a statistically significant trend (*p* = 0.470). Tigecycline resistance also varied across years with a significant difference (*p* < 0.001), although absolute resistance rates remained low. These findings indicate that resistance patterns in Bacteroides fragilis are dynamic and may be influenced by local antimicrobial usage practices and patient characteristics, highlighting the importance of continuous local surveillance [6, 7].

Despite its strengths, our study has limitations, including a relatively small sample size, which might lack statistical power, and reliance on data from a single institution. Additional long‐term prospective studies across multiple hospitals are necessary to examine a larger patient population and identify all potential risk factors. However, our study represents one of the few single‐center analyses focusing specifically on *B. fragilis* bacteremia, providing region‐specific clinical and microbiological data. In addition, the lack of molecular analysis limits our ability to fully characterize resistance mechanisms, particularly in relation to carbapenem resistance. Due to the unpredictable nature of antibiotic susceptibility in anaerobes, conducting antibiotic susceptibility tests is critically important. We anticipate that our data could assist clinicians in Türkiye and Europe, particularly in guiding empirical therapies where coverage for *B*. *fragilis* is necessary, such as in intra‐abdominal infections.

## 5. Conclusion

In conclusion, our study provides clinically relevant insights into the epidemiology, resistance patterns, and outcomes of *Bacteroides fragilis* bacteremia in a tertiary care setting. Our findings highlight the increased occurrence of *B. fragilis* bacteremia among patients with malignancies, possibly influenced by intensified anticancer therapies impacting the gastrointestinal barrier. Notably, our study identifies nosocomial acquisition and respiratory origin as independent risk factors for higher mortality in *B. fragilis* bacteremia. This emphasizes the importance of vigilance in healthcare settings, particularly in cases of nosocomial infections, which were associated with an extended hospitalization duration. The antibiotic resistance profiles revealed in our study raise concerns, especially regarding the traditionally effective antibiotics clindamycin and ampicillin. The emergence of resistance to carbapenems, although infrequent, underscores the evolving landscape of antibiotic susceptibility in *B*. *fragilis*. Metronidazole remains a reliable choice, showcasing a favorable susceptibility profile in our observed strains. These findings contribute to the understanding of *B. fragilis* bacteremia and may guide empirical treatment decisions, particularly in clinical settings where anaerobic coverage is required. Future studies integrating molecular diagnostics and multicenter data are warranted to better define resistance mechanisms and optimize management strategies.

## Author Contributions

Conceptualization, Enes Erul, Taha Koray Sahin, Meliha Çağla Sönmezer, and Murat Akova; methodology, Mervenur Demir Cuha, Nagihan Aka Türkmen, and Enes Erul; software, Nagihan Aka Türkmen, Mervenur Demir Cuha, Taha Koray Sahin, and Enes Erul; validation, Meliha Çağla Sönmezer, Taha Koray Sahin, Gülşen Hazırolan, and Murat Akova; formal analysis, Meliha Çağla Sönmezer, Enes Erul, and Taha Koray Sahin; investigation, Meliha Çağla Sönmezer, Mervenur Demir Cuha, Gülşen Hazırolan, and Enes Erul; resources, Mervenur Demir Cuha, Enes Erul, Taha Koray Sahin, Gülşen Hazırolan, Meliha Çağla Sönmezer, and Murat Akova; data curation, Enes Erul, Taha Koray Sahin, Meliha Çağla Sönmezer, and Murat Akova; writing–original draft preparation, Meliha Çağla Sönmezer, Enes Erul, Taha Koray Sahin, and Murat Akova; writing–review and editing, Meliha Çağla Sönmezer, Enes Erul, Taha Koray Sahin, and Murat Akova; visualization, Murat Akova and Meliha Çağla Sönmezer; supervision, Meliha Çağla Sönmezer and Murat Akova; project administration, Meliha Çağla Sönmezer and Murat Akova; funding acquisition, Meliha Çağla Sönmezer.

## Funding

This research received no external funding.

## Disclosure

All authors have read and agreed to the published version of the manuscript.

## Consent

Due to the retrospective nature of this study, informed consent was not obtained.

## Conflicts of Interest

The authors declare no conflicts of interest.

## Data Availability

The data that support the findings of this study are available from the corresponding author upon reasonable request. Due to patient privacy and ethical restrictions, they are not publicly available. The dataset underlying the congress abstract is also available.
